# Microbiome and ischemic stroke: A systematic review

**DOI:** 10.1371/journal.pone.0245038

**Published:** 2021-01-13

**Authors:** Yee Teng Lee, Nor Ismaliza Mohd Ismail, Loo Keat Wei

**Affiliations:** Department of Biological Science, Faculty of Science, Universiti Tunku Abdul Rahman, Kampar, Perak, Malaysia; University of Minnesota Twin Cities, UNITED STATES

## Abstract

**Background:**

Ischemic stroke is one of the non-communicable diseases that contribute to the significant number of deaths worldwide. However, the relationship between microbiome and ischemic stroke remained unknown. Hence, the objective of this study was to perform systematic review on the relationship between human microbiome and ischemic stroke.

**Methods:**

A systematic review on ischemic stroke was carried out for all articles obtained from databases until 22^nd^ October 2020. Main findings were extracted from all the eligible studies.

**Results:**

Eighteen eligible studies were included in the systematic review. These studies suggested that aging, inflammation, and different microbial compositions could contribute to ischemic stroke. Phyla Firmicutes and Bacteroidetes also appeared to manipulate post-stroke outcome. The important role of microbiota-derived short-chain fatty acids and trimethylamine N-oxide in ischemic stroke were also highlighted.

**Conclusions:**

This is the first systematic review that investigates the relationship between microbiome and ischemic stroke. Aging and inflammation contribute to differential microbial compositions and predispose individuals to ischemic stroke.

## Introduction

Ischemic stroke is one of the non-communicable diseases that contribute to the significant number of deaths worldwide as well as in Malaysia [1]. This multifactorial disease accords for 80% of stroke incidence annually [2], and is caused by various genetic-associated risk factors. Increasing evidences have shown that human microbiome is associated with ischemic stroke through the gut-brain axis. Human microbiome refers to the microbiota residing at the human body sites, including blood and gut. The bidirectional gut-brain axis connects the gut, the gut microbiota and the brain, when involves in the ischemic stroke pathophysiology [3–6]. Ischemic stroke alters the microbial composition in the gut, which affects the neurological outcomes subsequently [3–6].

Recent studies have suggested that gut microbiota, which is associated with obesity and diabetes mellitus, may trigger systemic inflammation, thereby modulating host inflammation for ischemic stroke pathogenesis [7,8]. Alternatively, aging weakens the immune system of the elderly and alters morphology and physiology of the gut, causing the elderly to have a different microbiome as compared to the young adults [9,10]. Meanwhile, mouse model coupled with aged microbiome possesses a slower recovery and a poorer functional outcome than that of the young microbiome following ischemic stroke event [11]. It has been shown that aging and inflammation manifest ischemic stroke occurrence in relation to human microbiome. Hence, the objective of this study was to examine the relationship between microbiome and ischemic stroke occurrence.

## Methods

### Literature search

This systematic review was adhered with PRISMA (Preferred Reporting Items for Systematic Reviews and Meta analyses) guideline [12]. Relevant articles published in English language, were retrieved from Pubmed, Scopus, Web of Science, Google Scholar and WPRIM databases [13]. The MESH-terms used include “microbiome”, “microbiota”, “ischemic stroke”, “cerebrovascular disease”, “cerebrovascular accident”, “brain ischemia”, “brain infarction”, “cerebral ischemia”, and “cerebral infarction”. Grey literatures and secondary references were also retrieved from the references cited in the articles, theses and dissertations, in order to determine if there is any additional eligible study. Animal and human studies reported on the association between microbiota/microbiome and ischemic stroke were included. Study design such as case-control, cohort and exploratory observational studies, published by any country were included. *In vitro* studies, commentaries, reviews and books were not considered. Human studies focused on neonates or pediatric were excluded as the pathophysiology of ischemic stroke is different from the adults. Data such as authors, study period, country of origin, study subject, sample size, mean age, gender, study design, sample source, method to determine microbiota, and main findings for each study were extracted by LYT and LKW. The extracted data were compared and compiled, and any disagreements were reconciled through discussion. The quality of the eligible studies was evaluated with NOS (Newcastle-Ottawa Scale). The last date of literature searching was 22^nd^ October 2020.

## Results

### Number of retrieved papers

**Fig 1:**
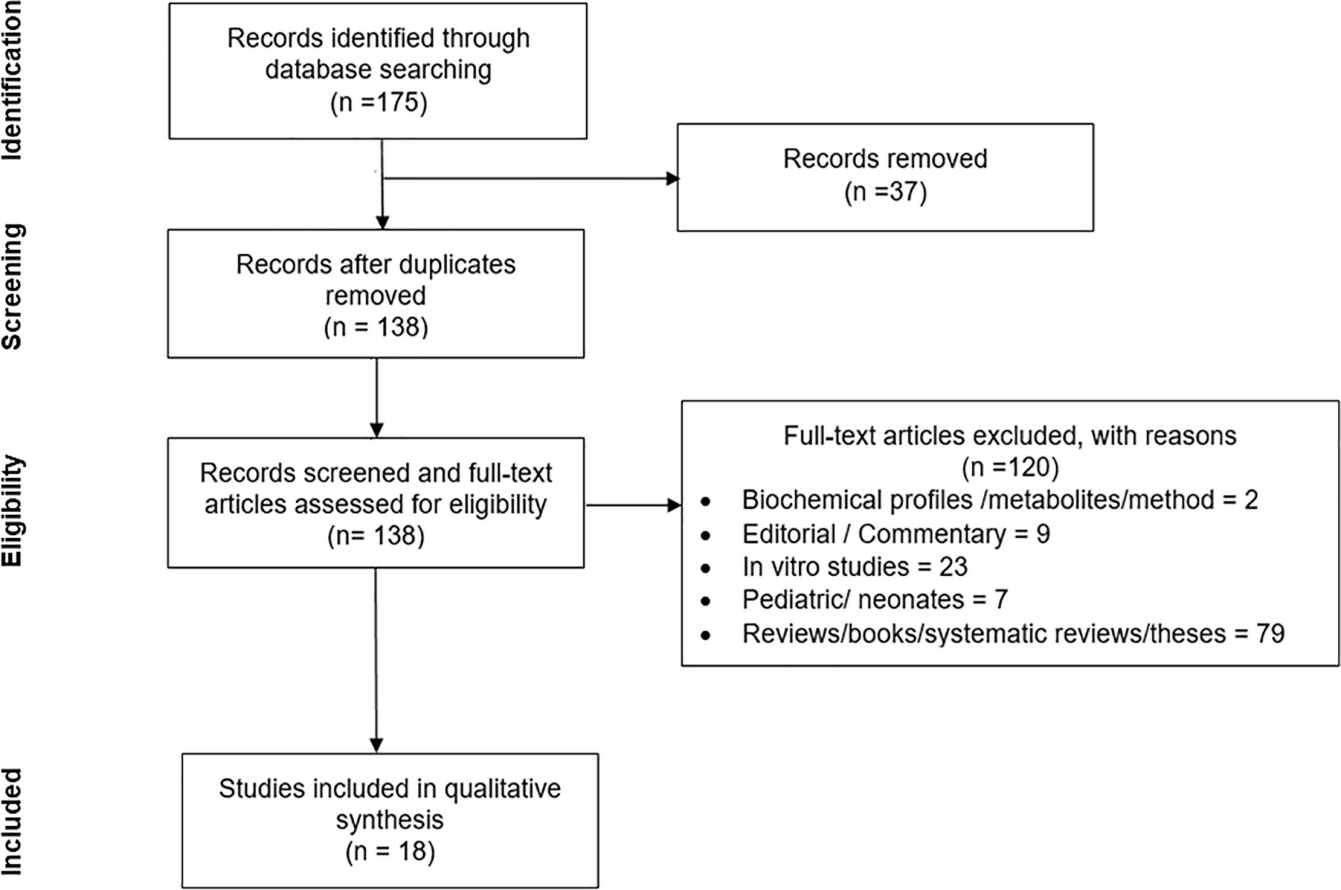
PRISMA flow diagram of the study selection process.

A total of 175 articles were obtained from the initial database searching. Following the removal of duplicates (n = 37), 138 full-text articles were evaluated and only eighteen relevant articles which fulfilled the inclusion and exclusion criteria were included in the final systematic review (Fig 1). Among the included studies, nine reported on human [14–18,20,24–26], seven were on animal [4–6,11,21–23] and two incorporated both human and animal subjects [3,19] (Table 1).

**Table 1:** Main findings of the eligible studies.

Author, year	Study Period	Country	Study subject	Total number		Age		Gender		Study design	Sample source	Method to determine microbiota	Main Findings
				**Case**	**Control**	**Case**	**Control**	**Case**	**Control**				
Haak et al, 2020 [14]	July 2010 –March 2014 (to recruit stroke patients); October 2017 –March 2018	Netherlands	Human	349	51	72 (62–80)^#^	71 (67–75)^#^	194 Caucasian Male*	29 Caucasian Male*	Case-control	Blood and feces (from rectal swabs)	Illumina MiSeq 16S rRNA amplicon sequencing on V3 region	1) A decrease in Firmicutes and Bacteroidetes while an increase in Proteobacteria were observed in stroke cases compared to controls. 2) The levels of *Escherichia/Shigella*, *Peptoniphilus*, *Ezakiella*, and *Enterococcus* were enriched in stroke cases. 3) An elevated abundance of *Blautia*, *Subdoligranulum*, and *Bacteroides* were shown in cases with transient ischemic stroke and controls. 4) A drastic reduction in *Anaerostipes*, *Ruminococcus*, and *Subdoligranulum* was observed in stroke cases. 5) No difference in Firmicutes/Bacterodetes ratio between stroke cases and controls. 6) Stroke cases exhibited remarkably levels of butyrate-producing bacteria compared to cases with transient ischemic attack and healthy controls. 7) Cases with ischemic stroke showed an increased abundance of TMAO-producing bacteria. 8) Cases with infection after stroke showed a reduced level of butyrate-producing bacteria compared to cases without post-stroke infection.
								155 Caucasian Female*	22 Caucasian Female*				
Tan et al, 2020 [15]	June 2017—January 2018	China	Human	140	92	59^#^	60^#^	95 Asian male*	51 Asian male*	Case-control	Blood and feces	Illumina HiSeq 2500 16S rRNA amplicon sequencing on V4 region	1) More than 80% of the intestinal microbiota were composed of families Bacteroidaceae, Ruminococcaceae, Enterobacteriaceae, Prevotellaceae, Lachnospiraceae, Veillonellaceae, Porphyromonadaceae, Verrucomicrobiaceae, Rikenellaceaea, Alcaligenaceae, Fusobacteriaceae, and Clostridiaceae. 2) A reduction in genera *Roseburia*, *Bacteroides*, *Lacnospiraceae*, *Feacalibacterium*, *Blautia*, and *Anaerostipes* were found in cases. 3) An increment in Lactobacillaceae, Akkermansia, Enterobacteriaceae, and Porphyromonadaceae were shown in cases. 4) The ratio of Firmicutes/Bacteroidetes was higher in cases. 5) The lack of microbiota-derived SCFAs was shown in cases, particularly those with severe stroke, leading to significantly poorer functional outcomes.
								45 Asian female*	41 Asian female*				
Li et al, 2020 [16]	-	China	Human	78	98	66.61 ± 12.07^¥^	64.01 ± 10.44^¥^	49 Asian male*	57 Asian male*	Case-control	Feces	Illumina MiSeq 16S rRNA pyrosequencing on V4 region	1) A reduction in Firmicutes and Bacteroidetes while an increased abundance of Proteobacteria and Actinobacteria were observed in cases. 2) Butyrate-producing genera *Faecalibacterium*, *Subdoligranulum*, *Eubacterium*, *Roseburia*, *Lachnoclostridium*, and *Butyricicoccus* were reduced remarkably in cases compared to controls. 3) The abundance of lactic-acid *Lactobacillus* and *Lactococcus* were higher in cases compared to controls. 4) Butyrate-producing bacteria were negatively associated with stroke severity, while lactic acid bacteria were positively associated with stroke severity. 5) A negative association between age and *Peptostreptococcaceae*, *Peptoclostridium*, and *Fusicatenibacter* was observed.
								29 Asian female*	41 Asian female*				
Huang et al, 2019 [17]	February 2018 –May 2018	China	Human	31	9	61 (40–94)^#^	61 (53–69)^#^	22 Asian male*	6 Asian male*	Case-control	Blood and feces	Illumina MiSeq 16S rRNA amplicon sequencing on V4 region	1) Abundance of *Blautia obeum* was relatively high in controls than in cases (p = 0.0441). 2) The abundance of *Streptococcus infantis* (p = 0.017) and *Prevotella copri* (p = 0.0099) were relatively higher in cases than in controls 3) *Blautia* genus was negatively associated with white blood cell count (r^2^ = 0.1053), while *Streptococcus* genus was found to be positively correlated with creatinine (r^2^ = 0.1328) and lipoprotein (r^2^ = 0.1004). 4) Bacterial pathways including methane metabolism (p = 0.0103), lipopolysaccharide synthesis (p = 0.0166 and p = 0.0249), bacterial secretion (p = 0.0156) and flagellar functions (p = 0.0065) were significantly expressed in the cases.
								9 Asian female*	3 Asian female*				
Li et al, 2019 [18]	May 2017 –January 2018	China	Human	30	30	60.47 ± 10.57^¥^	64.17 ± 12.67^¥^	21 Asian male*	18 Asian male*	Case-control	Blood and feces	Illumina MiSeq 16S rRNA amplicon sequencing on V1-V2 region	1) Higher abundance of *Odoribacter*, *Akkermansi*a, *Ruminococcaceae_UCG_005*, *norank_p_Flavobacteriaceae*, *norank_p_Parcubacteria*, and *Victivallis* were found in cases, while *Anaerostipes* and *Ruminiclostridium_5* were more abundant in controls. 2) Genera including *Odoribacter*, *Akkermansi*a, *Ruminococcaceae_UCG_005*, and *Victivallis* present in cases were producers for short-chain fatty acids. 3) *Bacteroides* (r = 0.42, p < 0.01) and *[Eubocterium]_rectole_group* (r = 0.336, p < 0.01) were positively correlated with LDL, while *Ruminococcus_2* (r = 0.381, p < 0.01) and *Lachnospiraceae_NK4A136_group* (r = 0.338, p < 0.01) were positively correlated with HDL. In contrast, *Enterobacter* (r = − 0.425, p < 0.01) was negatively correlated with HDL. 4) Genera *Christensenellaceae_R-7_group* and *norank_f_Ruminococcaceae* were positively correlated with stroke severity (r = 0.514, p < 0.05; r = 0.449, p < 0.05) and functional outcome (r = 0.471, p < 0.05; r = 0.503, p < 0.01). On the contrary, *Enterobacter* was negatively correlated with stroke severity (r = 0.449, p < 0.05) and functional outcome (r = 0.503, p < 0.01).
								9 Asian female*	12 female*				
Xia et al, 2019 [19]	February 2014 –February 2016 (to establish SDI model); January 2017 –December 2017 (Result validation for SDI model)	China	Human	187 (104 for SDI model; 83 for result validation)	160 (90 for SDI model; 70 for result validation)	18–80 [SDI model: 59.38 (12.61)^#^]	18–80 [SDI model: 56.62 (8.16)^#^]	SDI model: 78 Asian male*	SDI model: 73 Asian male*	Case-control	Feces	Illumina MiSeq 16S rRNA amplicon sequencing on V4 region	1) In SDI training cohort, a total of 18 genera of microbiota were significantly different between cases and controls, of which 7 of them were enriched in cases (*Coprococcus*, *Fecalibacterium*, *Haemophilus*, *Knoellia*, *Lachnospira*, *Prevotella*, *Roseburia*, *Unclassified Bradyrhizobiaceae*, *Unclassified Clostridiaceae*, *Unclassified Caulobacteraceae* and *Unclassified Erysipelotrichaeceae*), while another 11 genera were found exclusively in controls (*Bilophila*, *Butyricimonas*, *Oscillospira*, Parabacteroides, *Unclassified Enterobacteriaceae*, *Unclassified Rikenellaceae* and *Unclassified Ruminococcaceae*). 2) Positive correlation was observed between SDI and NIHSS as well as mRS, which made SDI a predictor for severe stroke and unfavorable early functional outcome in stroke. 3) An increase in (IL-17+) γδ T cells and a reduction in (CD4+CD25+) T cells were observed in the spleen of recipient mice that received high SDI feces after stroke, while an increased level of (IL-17+) γδ T cells and a decreased level of Treg (CD4+Foxp3+) cells were observed in the small intestines of the same group of recipient mice. The changes in the inflammatory cells might be associated with increased infarct volumes and neurological functional impairment.
									17 Asian female*				
								26 Asian female*					
			Mouse	20	20	6 weeks old	6 weeks old	Male	Male	*In vivo* animal experiment	Feces, spleens, intestinal intraepithelial lmyphocytes	Fecal transplantation; MCAO surgery	
Wang et al, 2018 [20]	May 2015 –March 2017	China	Human	10	10	53–82	53–82	-	-	Case-control	Blood and feces	Illumina HiSeq2500 16S rRNA sequencing on V4 region	1) The most abundant genera in cases were *Ruminococcus*, *Bacteroides*, *Prevotella*, *Parabacteroides*, *Dialister*, *Faecalibacterium*, *Megamonas*, *Roseburia*, and *Escherichia*. 2) A higher abundant of *Gammaproteobacteria* and a reduction in *Bacteroidia* were observed in cases. 3) APOE had a positive correlation with *Gammaproteobacteria* while it was negatively correlated with *Bacteroidia*.
Spychala et al, 2018 [21]	-	United States	Mouse	-	-	Young (8–12 weeks)	-	All male	-	*In vivo* animal experiment	Feces	MCAO surgery; Illumina MiSeq 16S rRNA amplicon sequencing on V4-V5 region; fecal transplantation; short-chain fatty acids (SCFA) analysis	1) Stroke increased the ratio of phyla Firmicutes and Bacteroidetes through dysbiosis in both young and aged mice, yet, the Firmicutes/Bacteroidetes ratio was significantly 9-fold higher in aged mice than young mice. 2) Young microbiome recipient mice showed lower Firmicutes/Bacteroidetes ratio and improved stroke recovery; conversely, aged microbiome recipient mice showed a higher Firmicutes/Bacteroidetes ratio and did not fully recover from neurological deficits. 3) A significantly higher mortality rate was seen in aged microbiome recipient mice. 4) Higher trend of cytokines and short-chain fatty acids were observed in young microbiome.
						Old (18–20 months)							
Singh et al, 2018 [22]	-	Germany	Mouse	-	-	10 to 12 weeks old	10 to 12 weeks old	All female	All female	*In vivo* animal experiment	Feces, intestinal mucose	cMCAO surgery, Illumina MiSeq 16S rRNA amplicon sequencing on V1-V3 region	1) A reduced infact volume (p < 0.05) and a high number of microglia/macrophages (p < 0.05) were observed in mice colonized with bacteria after stroke. 2) Massive activation of microglia which caused higher sphericity index (p < 0.05) and reduced ramifications (p < 0.05) in the colonized mice had been observed in peri-infact area, and it increased the cerebral expression of pro-inflammatory cytokines, including IL-1β (p < 0.01) and TNF-α (p < 0.05). 3) An increased in lymphocytes, including T and B cells, were observed in the spleens, intestines, and the brain of the colonized mice.
Stanley et al, 2018 [23]	-	Australia	Mouse	-	-	7 to 10 weeks old	7 to 10 weeks old	All male	All male	*In vivo* animal experiment	Intestinal mucosa, i.e. Gut, including duodenum, jejunum, ileum, cecum and colon	MCAO surgery; Illumina MiSeq 16S rRNA amplicon sequencing on V3-V4 region	1) Microbial compositions in the mucosa of gastrointestinal tract were significantly different between sham-operated and post-stroke mice. 2) Abundance of *Clostridium* species and *Akkermansia muciniphila* were significantly higher in post-stroke mice compared to sham-operated mice. 3) The stroke-induced change in microbial composition altered the predicted functional potential of microbiota by upregulating 20.5% of 39 KEGG pathways including pathways associated with infectious diseases, membrane transport and xenobiotic degradation.
Boaden et al., 2017 [24]	July 2012 –April 2013	England	Human	50	-	80.5 (65–86)^#^	NA	24 Caucasian Male*	NA	Cross sectional	Salica swab (Oral cavity including buccal mucosa, tongue, gingiva and hard palate)	TOPO cloning and 16s rRNA gene sequencing	1) 103 bacterial phylotypes were found, of which 65% were Gram positive, 33% were Gram negative and 2% were Gram variable. 2) 20 most common bacterial phylotypes included *Streptococcus* species (n = 14), Gram-negative *Veillonella* species (n = 3), Gram-positive *Rothia mucilaginosa* (n = 1), Gram-negative *Treponema pedis* (n = 1) and Gram-positive *Lactobacillus fermentum* (n = 1). 3) 30% of the acute stroke cases were diagnosed with at least one infection.
								26 Caucasian Female*					
Yamashiro et al, 2017 [25]	April 2014 –March 2015	Japan	Human	41	40	65.4 ± 14.1^¥^	67.4 ± 8.9^¥^	31 Asian male*	24 Asian male*	Case-control	Blood and feces	16S and 23S rRNA-targeted quantitative reverse transcription (qRT)-PCR; high-performance liquid chromatography analyses on blood	1) Gut microbiota were present in the blood of 7.5% controls and 4.9% cases. 2) Abundance of *Lactobacillus ruminis* was significantly higher in cases and it was positively correlated with level of interleukin-6 (IL-6) in serum. 3) The increased abundance of *Atopobium* cluster and *L*. *ruminis* as well as the reduced abundance of *L*. *sakei* subgroup were significantly associated with ischemic stroke. 4) The reduced abundance in *Clostridium coccoides* was associated with Type II diabetes (p = 0.01), and it was negatively correlated with levels of IL-6 (p < 0.01), hsCRP (p < 0.05), HbA1c level (p < 0.05) and LDL-cholesterol (p < 0.05). 5) Ischemic stroke was closely associated with low organic acid concentrations (p = 0.02), including low acetic acids (p = 0.003) and high valeric acids (p = 0.003).
								10 Asian female*	16 Asian female*				
Stanley et al., 2016 [3]	-	Australia	Human	36	10	65.5 (Without infection)	63–71	22 Caucasian Male*	10 Caucasian Male*	Case-control	Blood, urine, lung tissues, and sputum	Illumina MiSeq 16S rRNA amplicon sequencing on V3-V4 region	1) More than 60% of the microbiota in the lungs were proven to originate from small intestines significantly (p = 0.01). 2) Stroke reduced the gut barrier permeability and caused intestinal dysfunction in order to allow the passage of microbiota into peripheral tissues.
						76.5 (With infection)		14 Caucasian Female*					
			Mouse	-	-	7 to 10 weeks old	7 to 10 weeks old	All male	All male	*In vivo* animal experiment	Lung tissues, gut, liver and skin	MCAO surgery	
Benakis et al., 2016 [4]	-	United States	Mouse	-	-	6 to 8 weeks old	-	All male	-	*In vivo* animal experiment	Feces	MCAO surgery; Illumina MiSeq 16S rRNA amplicon sequencing on V4-V5 region	1) Antibiotic-induced dysbiosis caused a change in the intestinal microbiome and affected the immune system in stroke. 2) An increase in intestinal Treg cells (regulatory T cells) and a reduction in IL-17+ γδ T cells was observed, showing the trafficking of effector T cells from the gut to leptomenings in order to enhance ischemic neuroinflammation. 3) Reduction of Bacteroidetes and increase in Proteobacteria in the gut flora might provide neuroprotection to the mice from brain injury.
Singh et al., 2016 [5]	-	Germany	Mouse	-	-	-	-	All male	All male	*In vivo* animal experiment	Feces	MCAO surgery; Illumina MiSeq 16S rRNA amplicon sequencing on V1-V3 region	1) Ischemic stroke induced dysbiosis of microbiota from Peyer’s patches to the injured brain by reducing species diversity and increasing growth of Bacteroidetes. 2) Results of fecal microbiota transplantation proved the dysbiosis of Thelper cells from the intestines to the brain, where Treg cells were increased in order to reduce the inflammatory collateral damage.
Crapser et al., 2016 [6]	-	United States	Mouse	-	-	Young (8–12 weeks old)	-	All male	-	*In vivo* animal experiment	Mesentric lymph nodes (MLNs), spleens, liver, gut (intestines), lung tissues	MCAO surgery; 16s rRNA gene sequencing	1) Higher number of gut microbiota was observed in the livers and lungs of aged stroke mice. 2) Ischemic stroke enhanced gut permeability and induced gut dysbiosis in both young and aged mice. 3) *Escherichia* species was found in young stroke mice only while *Enterobacter* species could only be detected in aged stroke mice. 4) Higher gut permeability was observed in aged mice, and it led to high neurological deficits and higher mortality rate in aged mice after stroke. 5) Greater bacterial burden was observed in MLNs and other peripheral organs of aged stroke mice, causing aged stroke mice developed sepsis at a higher risk.
						Old (18–20 months old)							
Yin et al, 2015 [26]	February 2014 –February 2015	China	Human	322 (322 contributed blood samples; 141 contributed fecal samples)	231 (231 contributed blood samples; 94 contributed feces samples)	61 (19)^#^	56 (11)^#^	220 Asian male*	130 Asian male*	Case-control	Blood and feces	Illumina MiSeq 16s rRNA amplicon sequencing on V4 region	1) Majority of the gut microbiota were of phyla Bacteroidetes, Firmicutes, and Proteobacteria. 2) 80% of the gut microbiome were mainly *Bacteroides*, *Prevotella*, *Faecalibacterium*, *Escherichia*/*Shigella*, and *Roseburia*. 3) A higher abundance of Proteobacteria and a lower abundance of *Bacteroides*, *Prevotella* and *Faecalibacterium* were observed in cases. 4) A significant lower TMAO levels were observed in cases.
								102 Asian female*	101 Asian female*				
Jandzinski, 2015 [11]	-	United States	Mouse	48 mice	-	Young (2 months old) (young)	-	All male	-	*In vivo* animal experiment	Feces	MCAO; Fecal transplantation	1) More than 90% of gut microbiota were of phyla Bacteroidetes and Firmicutes. 2) High Bacteroidetes / Firmicutes ratio and larger portions of Verrucomicrobia were observed in feces of young mice. 3) High Firmicutes / Bacteroidetes ratio and presence of Deferribacteres were detected in feces of old mice.
						Old (16 months old)							

-, not stated in the primary study

*, gender for human subjects only; NA, not applicable; MCAO, Middle cerebral artery occlusion; SDI, Stroke Dysbiosis Index; NIHSS, National Institutes of Health Stroke Scale; mRS, Modified Rankin Scale.

^#^, Data were presented as median (interquartile range)

^¥^, Data were presented as mean ± standard deviation.

### Characteristics of included studies

#### Animal model studies

All seven animal studies focused on gut microbiome of mouse [4–6,11,21–23]. The common mouse model found in all included animal studies was a C57BL/6 mouse model, however, Jandzinski [11] did not declare which mouse model was used to show the effect of age of microbiome on ischemic stroke.

Benakis et al [4] utilized multiple mouse models for different analyses, including wild-type C57BL/6, Il10^−/−^, Il17a^−/−^, Il17a-eGFP, Trdc-eGFP and KikGR33 mice. They utilized antibiotics treatment to uncover the neuroprotective effect of altered microbial composition on ischemic injury and examine the stroke-induced immune response using brain tissues, blood, and other body tissues including spleens, lymph nodes, and intestinal cells from the mice [4].

Besides, two studies used wild-type C57BL/6J and Rag1^−/−^ male mice as well as germ-free (GF) C57BL/6J and GF Rag1^−/−^ female mice [5,22]. Both studies aimed to examine neuroinflammatory response after stroke dysbiosis. Another two studies employed C57BL/6 mice to compare both young and aged microbiome [6,21], whereas another study used the same C57BL/6J mouse model to uncover the microbial composition in intestinal mucosal after stroke [23].

#### Clinical studies

All the nine clinical studies reported on gut microbiome, where most of the clinical studies were conducted in both case and control cohorts [14–18,20,25,26], except that Boaden et al [24] who studied on stroke patients. All studies collected fecal samples from the study subjects for microbiome analyses and blood samples for blood biochemical assays, except that Li et al [16] collected the fecal samples and Boaden et al [24] collected the saliva sample and swabs within the oral cavity of stroke cases.

#### Studies reporting on both human and animal subjects

Two studies were conducted on both human and animal subjects [3,19]. One study reported the presence of microbiota in multiple human body sites, such as blood, urine and sputum of human, as well as lung and gut of mouse [3]. This study recruited arterial ischemic stroke cases and used a C57BL/6J mouse model to conduct the microbial analysis of ischemic stroke [3]. Xia et al [19] studied about gut microbiome by collecting human feces as well as mouse feces. This study collected fecal samples from large-artery atherosclerotic cases and healthy controls to examine the microbial composition and establish stroke dysbiosis index. The same study also included a male C57BL/6 mouse model to conduct fecal transplantation experiment and neurobehavioral examination to assess the effect of post-stroke dysbiosis [19].

### Main outcome of eligible studies

#### Microbiome

Eight phyla of bacteria namely Firmicutes, Bacteroidetes, Proteobacteria, Actinobacteria, Spirochaetes, Deferribacteraceae, Verrucomicrobia and Tenericutes were commonly detected in these 16 studies, with both phyla Firmicutes and Bacteroidetes predominated the microbial composition in both human and mice [3–6,11,14–26].

Singh et al [5] showed that phyla Firmicutes and Bacteroidetes made up the major composition of mice gut microbiome, followed by Actinobacteria. This was supported by the other two studies which demonstrated that more than 90% of the fecal microbiome were consisted of phyla Bacteroidetes and Firmicutes [11,21]. Another study revealed that the antibiotic treatment increased the abundance of Proteobacteria while reducing the Firmicutes and Bacteroidetes in antibiotic-sensitive mice [4]. It also demonstrated that phyla Firmicutes and Bacteroidetes were the major gut microbial composition in antibiotic-resistant mice, while Proteobacteria was predominantly found in the antibiotic-sensitive mice [4].

An animal study observed that the organs of mice were colonized by *Staphylococcus* and *Enterococcus* after ischemic stroke [6]. Another study found that genera *Lactobacilus*, *Actinomyces*, *Ruminococcus*, Unknown Peptostreptococcaceae, *Clostridium*, *Brevundimonas*, and *Eshcherichia* and *Shigella* were more abundantly available in the lung of post-stroke mice as compared to the control group. More specifically, the relative abundance of *Escherichia* and *Shigella* species, *Streptococcus* species, *Lactobacillus* species, *Brevundimoas nasdae* and *Staphylococcus sciuri* were significantly higher in the lungs of post-stroke mice [3]. A subsequent study published by the same group of researchers showed an elevation in multiple *Clostridium* species, *Parabacteroides goldsteinii*, *Anaerotruncus colihominis*, *Alistipes shahii*, *Akkermansia muciniphila* and *Roseburia intestinalis* in the gastrointestinal tract of post-stroke mice [23].

The clinical studies found that the abundance of phylum Firmicutes is an independent predictor for ischemic stroke risk [16–18,25]. Particularly, genera *Streptococcus*, *Lactobacillus and Prevotella* were descent from phylum Firmicutes prevailed among ischemic stroke cases [16–18,25]. Boaden et al [24] found that Streptococcus species were the most abundant microbiota that made up 70% of the oral microbial community in stroke patients. A study showed that the abundance of *S*. *infantis* and *P*. *copri* were found higher in cases with ischemic stroke, while *Blautia obeum* was relatively lower among ischemic stroke cases [17]. The lactic acid-producing *Lactobacillus* and *Lactococcus* were significantly enhanced in fecal samples of cases [16]. In addition, Wang et al [20] observed an increase in *Gammaproteobacteria* and a decrease in *Bacteroidia* in cases.

However, another study reported that Proteobacteria was increased in the cases, and a lower abundance of *Bacteroides*, *Prevotella* and *Faecalibacterium* was observed [26]. This study also demonstrated a higher abundance of Proteobacteria and a reduction of Bacteroides in cases following more severe stroke outcomes [26]. Similarly, another study by Haak et al [14] also observed that the abundance of Proteobacteria was increased while the levels of Firmicutes and Bacteroidetes were reduced in stroke patients. Li et al [16] observed that the relative abundance of genera *Odoribacter*, *Akkermansi*a and *Ruminococcaceae_UCG_005* were significantly higher in stroke cases. More specifically, *Ruminococcaceae_UCG_005* was enhanced in severe stroke cases [18]. Tan et al [15] also reported an increase in *Akkermansia* along with *Lactobacillaceae*, *Enterobacteriacea* and *Porphyromonadaceae* in cases with acute ischemic stroke, particularly those with severe stroke. Another clinical study demonstrated that ischemic stroke was independently associated with reduced amount of *L*. *sakei* subgroup and increased abundance of *Atopobium* cluster and *L*. *ruminis* [25].

A study by Stanley et al [3] identified *Enterococcus* spp., *Escherichia coli* and *Morganella morganii* from the blood, urine or sputum samples from 22.2% of stroke cases, and there was no culturable microbiota in the control group. Meanwhile, Haak et al [14] observed that the abundance of aerobic *Enterococcus* and *Escherichia/Shigella* were increased in stroke patients, while a drastic reduction in obligate anaerobic *Anaerostipes*, *Ruminococcus*, and *Subdoligranulum* was reported in stroke patients. Xia et al [19] found that seven genera were significantly enriched in the fecal microbial composition of stroke cases, such as *Butyricimonas*, *Parabacteroides*, Unknown Rikenellaceae, Unknown Ruminococcaceae, *Oscillospira*, *Bilophila* and Unknown Enterobacteriaceae.

#### Diversity analyses

A total of nine eligible studies measured α-diversity [3–5,14,16–18,20,22,26]. Two studies reported no significant difference between post-stroke mice and sham-operated mice [3,22]. Similar phenomenon was also observed between stroke cases and healthy controls in the other three clinical studies [16–18]. On the other hand, Singh et al [5] reported a reduced microbial diversity in the gut microbiome of post-stroke mice as compared to the controls, whereas Benakis et al [4] observed a reduced α-diversity in antibiotic-sensitive mice. Similarly, two clinical study also reported a lower microbial diversity in cases with ischemic stroke when compared to healthy controls [14,20]. In contrast, Yin et al [26] showed a significantly higher microbial diversity in cases than that of the controls.

There are ten studies measured β-diversity, including three animal studies [3,5,22] and seven human studies [14–16,18–20,26]. Eight studies demonstrated significant differences in β-diversity between stroke group and control groups [3,5,14,15,18–20,22,26], while one study reported no difference in the microbiota structure between both cases and controls [16]. Interestingly, a study showed that cases with lower SDI index exhibited quite similar fecal microbial composition as the healthy controls [19].

#### Effect of aging on microbiome and ischemic stroke

Three studies assessed the impact of age on microbial composition [6,11,21], among which two evaluated the role of age in the gut microbiome of stroke mouse model, particularly on Firmicutes/Bacteroidetes ratio [11,21]. Two studies observed a higher Firmicutes/Bacteroidetes ratio in aged microbiome of the mice when compared to the young microbiome following a stroke event [11,21]. Ischemic stroke induced the increment of Firmicutes and reduced the abundance of Bacteroidetes in both young and aged mice, but the Firmicutes/Bacteroidetes ratio was greatly enhanced in aged mice when compared to the young mice [21]. These studies also found that the aged mice with a high Firmicutes/Bacteroidetes ratio were unable to recover from neurological deficits and showed a higher mortality rate [11,21].

A study showed a significant higher bacterial burden in mesenteric lymph nodes of aged mice as compared to young mice after stroke dysbiosis, which caused the aged mice to have a higher mortality rate [6]. The same study also identified genus *Escherichia* in young mice only, while the presence of *Enterobacter* could only be found in aged mice [6]. In addition, Jandzinski [11] also showed that phylum Deferribacteres was detected in aged microbiome only, while only young microbiome showed the presence of Verrucomicrobia.

#### Stroke dysbiosis

Ten included studies suggested that stroke induced gut dysbiosis, altered the microbial composition, and manipulated the post-stroke outcome [3–6,11,15,16,19,21,26]. Crapser et al [6] proved that ischemic stroke increased gut permeability, induced bacterial translocation from the gut to mesenteric lymph nodes, spleens, livers and lungs, and led to a high risk of infection due to gut dysbiosis. A study demonstrated that a significant depletion of microbiota was seen in the intestinal compartments including ileum and colon after stroke, while a remarkable increase of microbiota could be observed in the lung of post-stroke mice [3]. The same animal study also proved that the microbiota dysbiosis was induced by stroke, which subsequently impaired the immune and barrier defense system of the host body [3]. Singh et al [5] discovered that the species abundance in Firmicutes, Bacteroidetes and Actinobacteria were modulated by gut dysbiosis after stroke.

Tan et al [15] found a reduction of SCFAs-producing bacteria in cases with ischemic stroke when compared to controls. Nonetheless, a study showed that the butyrate-producing bacteria were remarkably less abundant in ischemic stroke and with increasing abundance of lactic acid bacteria [16]. Besides, Yin et al [26] determined more opportunistic pathogens in cases which were associated with significant higher TMAO levels. Meanwhile, two studies observed a shift in Firmicutes/Bacteroidetes ratio in the fecal microbial composition of mice as an effect of stroke dysbiosis, where ischemic stroke significantly increased the ratio Firmicutes/Bacteroidetes [11,21].

Two studies demonstrated gut dysbiosis through the translocation of intestinal immune cells to the brain after stroke [4,5]. A study observed that T cells migrated from the intestinal lamina propria to the meninges after stroke [4], whereas another study showed that lymphocytes could migrate from Payer’s Patches to the brain after induced by stroke [5]. Intriguingly, a study established a stroke dysbiosis index (SDI), a microbiota index in ischemic stroke based on the gut dysbiosis pattern, whereby the higher the SDI index, the more severe the brain injury and the poorer functional outcome the cases had. The same study also observed that genera *Oscillospira*, *Enterobacteriaceae*, *Bacteroides*, and *Bacteroidaceae* were more abundant in ischemic stroke cases with high SDI (SDI-H) [19].

#### Neuroinflammatory response by microbiota after ischemic stroke

A study showed that stroke elevated gut permeability and selectively increased vascular permeability in the jejunum and ileum of the post-stroke mice, leading to a significant increase in the abundance of goblet cells in the jejunum and ileum [3]. A significantly increased abundance of IgA^+^ B cells were observed in the mesenteric lymph nodes of the post-stroke mice, while a significant reduction of neuronal submucosal cholinergic ChAT^+^ cells was observed in post-stroke mice [3].

A recent study by Xia et al [19] assessed potential microbiota dysbiotic effect on stroke injury in mouse model by performing fecal transplantation from stroke cases with SDI-H to mice. An aggravated abundance of pro-inflammatory (IL-17^+^) γβ T cells was seen in both spleen and small intestine of the SDI-H recipient mice, whereas depleted (CD4^+^CD25^+^) helper T (T_helper_) cells and regulatory T cells (T_reg_) (CD4^+^ Foxp3^+^) cells were observed in the spleen and small intestine of the SDI-H recipient mice. The results demonstrated an elevated infarct volume and poorer neurological functional outcome in the SDI-H recipient mice after stroke [19].

Another study demonstrated the neuroprotective effect of gut microbiota on the ischemic injury by colonizing the germ-free mice with gut microbiota [22]. As a result, a higher number of microglia/macrophages as well as a remarkable increased expression of proinflammatory cytokines were observed in the ischemic brain of the post-stroke mice [22]. The cell counts of T_helper_, T_reg_ and Th17 were increased in the Peyer’s patches and even enhanced in the spleens after stroke, while the similar pattern was also observed in the ischemic brain, leading to a smaller lesion volume in the mice brain after stroke [22].

Crapser et al [6] demonstrated that a significantly higher percentage of CD4^+^ and CD8^+^ T cells as well as the event of lymphopenia were found in the blood of stroke mice as compared to sham-operated mice, while a remarkably greater proportion of infiltration leukocytes, including CD3^+^ T cells were observed in the brain of stroke mice. Other than that, Yamashiro et al [25] showed that *L*. *ruminis* was positively correlated with interleukin-6 (IL-6) level, while *C*. *coccoides* was negatively correlated with IL-6 and high sensitivity C-reactive protein (hsCRP).

Furthermore, mice receiving young microbiome possessed much higher levels of IL-4 and granulocyte-colony stimulating factor (G-CSF), while the elevation of levels of IL-6, tumor necrosis factor alpha (TNF-α), Eotaxin, and CCL5 were shown in the mice with aged microbiota after stroke [21]. Crapser et al [6] showed that IL-6 levels were generally increased in both young and aged mice after stroke, while aged mice showed a much higher level of IL-6. A significant negative correlation was observed between the effects of aging and serum lipopolysaccharide-binding protein (LBP) levels, causing a higher rate of sepsis in aged mice due to a lower level of immune response [6].

Interestingly, a study investigated the neuroprotection of the altered gut microbiome after ischemic injury by treating a group of mice (AC^Sens^) with antibiotics amoxicillin and clavulanic acid (AC) [4]. An elevated cell count of T_reg_ and a depletion in IL-17^+^ γβ T cells were observed in the AC^Sens^ mice, while a higher number of IL-17^+^ γβ T cells were in the meninges after stroke. Besides, the study also found that gut microbiota was able to manipulate the function of dendritic cell in the intestine, as it could suppress the differentiation of IL-17^+^ γβ T cells with the help of IL-10 [4].

#### KEGG pathway analysis

Only three studies performed KEGG functional pathway analysis [16,17,22]. Huang et al [17] showed that four bacterial pathways were present in the cases with ischemic stroke, of which, lipopolysaccharide synthesis was significantly enriched in the cases with ischemic stroke. Another study reported that a total of eight KEGG pathways were found to be significantly upregulated in post-stroke mice as compared to sham-operated mice [22]. Intriguingly, both studies observed that bacterial secretion was significantly enhanced in stroke group when compared to the control group. In contrast, an enhancement in human disease-associated module including genes corresponding to infectious diseases was observed in cases with ischemic stroke [16]. The same study also observed a significant reduction of the sporulation functional gene expression level of butyrate-producing bacteria while a remarkable increased expression of the lactic acid bacteria-related phototransferase system in cases with ischemic stroke [16].

#### Association between clinical markers and gut microbiota in ischemic stroke

Huang et al [17] found that cases with ischemic stroke exhibited significantly higher levels of hyperlipidemia, total cholesterol, triglycerides, higher blood pressure and white blood cell count. Besides, Xia et al [19] also showed a positive association between alcoholic consumption and HbA1c with severe stroke, where age, creatinine and uric acid were negatively correlated with post-stroke functional outcomes. Besides, Xia et al [19] also found that stroke dysbiosis and white blood cell count were the independent predictors of severe stroke, while stroke dysbiosis was found to be an independent predictor of early poor functional outcome after stroke.

While Huang et al [17] found no difference in blood glucose level, creatinine and uric acid between cases and controls, Xia et al [19] showed a positive association between blood glucose, creatinine and uric acid with severe stroke. A study also found a positive correlation between uric acid and *Dialister*, while blood glucose was remarkably negative correlated with genera *Ruminococcaceae_*UCG-002, *Alistipes* and *Ruminococcus_1* [18]. While Yamashiro et al [25] found that type 2 diabetes was associated with a lower level of *Clostridium coccoides*, Xia et al [19] showed that diabetes mellitus was positively related to severe stroke and negatively correlated with functional recovery after stroke.

Significant higher levels of lipoprotein and high-density lipoprotein (HDL) were demonstrated in cases with ischemic stroke [17], while the lipoprotein levels between cases and controls were similar in another study [14]. A study showed a significant positive correlation between HDL and genera *Ruminococcus_1*, *Ruminococcus_2* and *Lachnospiraceae_NK4A136_group*, while a negative correlation was found between HDL and *Enterobacter* [18]. In addition, Li et al [18] also unveiled a notably positive correlation between LDL and *Bacteroides* and *Eubacterium rectole* group. Meanwhile, LDL was negatively correlated with norank_O_Mollicutes_RF9 and *C*. *coccoides* [18,25]. Besides, Wang et al [20] showed that Bacteroidia was negatively correlated with apoliprotein E (APOE), while Gammaproteobacteria was positively correlated with APOE level. Intriguingly, a positive correlation was also shown between homocysteine and genera *Megamonas* and *Fusobacterium* [18].

#### Microbiota-derived metabolites and ischemic stroke

Of the 16 studies, eight studies observed the association between microbiota-derived metabolites, such as short-chain fatty acids (SCFAs) and trimethylamine-N-oxide (TMAO), and ischemic stroke [14–16,18,21,23,25,26]. Only two studies reported on the association between TMAO level and ischemic stroke [14,26]. One study showed a remarkable decrease in blood TMAO levels in cases with ischemic stroke after gut dysbiosis [26], while another study reported a higher level of TMAO-producing microbiota in ischemic stroke cases, especially in ischemic stroke cases with severe stroke [14]. When comparing to the healthy controls, the median concentration of total SCFAs was lower in cases, especially those who exhibited poorer neurological outcomes [15]. The concentration of acetate was significantly lower in cases [25]. Meanwhile, valerate and isovalerate were remarkably higher in cases [25]. The study also observed a negative correlation between the concentrations of acetate and propionate with the levels of HbA1c and LDL, while the concentration of valerate was positively associated with the WBC counts and the level of hsCRP [25]. Furthermore, a study showed that acetate and propionate were significantly reduced in mice with aged microbiomes as compared to young microbiome [21].

Three studies demonstrated a reduction of SCFA-producing bacteria in cases with ischemic stroke [14–16]. However, a significantly higher abundance of SCFAs-producing genera *Odoribacter* and *Akkermansia* were found in the gut of ischemic stroke cases [18]. Similarly, as high as two-fold abundance of *Akkermansia* in the intestinal mucosa and lungs of stroke mice has been observed [23]. Besides, the concentrations of acetate and propionate were reduced by approximately 68% in mice with aged microbiomes as compared to mice with young microbiomes.

## Discussion

To our knowledge, this is the first study that comprehensively assessed the relationship between microbiome and ischemic stroke via systematic literature searching. This study provides a critical summary of the available evidence regarding the potential role of human microbiome in ischemic stroke.

In human microbiome, Firmicutes and Bacteroidetes are the main phyla that predominate more than 90% of the microbial composition. Across the studies, Firmicutes appears to be the most significant phylum present in ischemic stroke. For instance, genera *Lactobacillus* and *Streptococcus* were highly associated with ischemic stroke in the gut of both mice and ischemic stroke cases. *Lactobacillus* contributes to the folate synthesis, which generates coenzyme to participate in one carbon metabolism [27]. This phenomenon helps in maintaining the balance in levels between methionine and homocysteine, which is highly associated with neurodegenerative diseases including ischemic stroke [27,28]. Besides, a high level of creatinine in serum has been suggested as a predictor for mortality rate of stroke [29], and cases with elevated creatinine level within 48 hours of stroke injury might have a higher risk of developing acute kidney injury [30].

The correlation between APOE and *Gammaproteobacteria* and *Bacteroidia* was reported in one study [20]. The APOE produces lipoprotein and gets involved in the fat metabolism. Lipoprotein also appears to be a risk factor of ischemic stroke [31], and a strong positive association is suggested between lipoprotein and young stroke cases [32]. TMAO is a microbiota-derived product that promotes atherosclerosis, leading to the development of stroke and cardiovascular diseases [33]. The negative correlation between TMAO and ischemic stroke shown in Yin et al [26] contradicted with another study that demonstrated a positive association between the increased TMAO level and enhanced stroke severity [14,34]. However, a recent study showed that the TMAO level was higher in cases with ischemic stroke on admission before a remarkable reduction in TMAO level was observed after 48 hours [34]. It was suggested that the decrease in TMAO level could be due to gut dysbiosis after ischemic stroke, which might aid in improving the recovery following ischemic stroke event [26].

A study reported the ability of antibiotic modulation in the gut microbiome in improving the ischemic injury in the brain [4]. With the alteration in the microbial composition in antibiotic-sensitive mice, antibiotics was able to reduce the infarct volume while improving recovery outcome in antibiotic-sensitive mice after stroke [4]. In fact, antibiotics treatments have been given to the cases with ischemic stroke for post-stroke infections. A total of three meta-analyses have shown that preventive antibiotic treatment after stroke onset can reduce the infections of post-stroke cases significantly, including the urinary tract infections [35] and pneumonia [36]. Nonetheless, none of these studies reported that the use of antibiotics reduces post-stroke functional outcome of the cases [35–37]. Therefore, the role of antibiotics in improving recovery outcome in ischemic stroke remains unconfirmed.

The association between Firmicutes/Bacteroidetes ratio and ischemic stroke appears to be confirmed in mice models. An increment of Firmicutes/Bacteroidetes ratio is the hallmarks of aging and dysbiosis [21,38]. A high Firmicutes/Bacteroidetes ratio worsened the neurological deficits of the mice after ischemic stroke and can increase the mortality rate [11,21]. In fact, Firmicutes/Bacteroidetes ratio has been associated with various risk factors of ischemic stroke, including hypertension [39,40], obesity [41,42], and diabetes mellitus [43]. Besides, the present systematic review also observed the impact of aging in ischemic stroke. Two studies demonstrated that a higher Firmicutes/Bacteroidetes ratio was present in an aged microbiome of mice, which then aggravated the post-stroke outcome [11,21]. However, only two clinical studies measured the Firmicutes/Bacteroidetes ratio in cases with ischemic stroke [14,15]. While Tan et al [15] demonstrated a higher Firmicutes/Bacteroidetes ratio in cases with acute ischemic stroke, Haak et al [14] reported no difference in Firmicutes/Bacteroidetes ratio between cases and controls. In fact, a recent study showed that Firmicutes/Bacteroidetes ratio increased with age in healthy subject [44]. The study observed Firmicutes was significantly 40% higher in elderly (60–69 years group) as compared to children (0–9 years group), while Bacteroidetes tended to be remarkably 80% lower in the elderly [44].

Other than modulating Firmicutes/Bacteroidetes ratio, aging has been shown to exert significant changes to microbial composition. It was observed that phylum Verrucomicrobia was present exclusively in young microbiome of stroke mouse model. Particularly, the SCFA-producing *Akkermansia* under phylum Verrucomicrobia has been observed in ischemic stroke. SCFAs are important metabolites in intestinal homeostasis, as it helps to strengthen the gut barrier function and generate intestinal epithelial cells [45]. It also helps in increasing the expression of tight junction protein and strengthening blood-brain barrier, thus improving the ischemic injury in the brain [27]. Therefore, the present study suggested that the presence of SCFAs might be the reason why mice with young microbiome recovered faster and better as compared to mice that contained aged microbiome.

About 39% of the studies reported that metabolic role of SCFA-producing microbiota is crucial in the recovery of ischemic stroke. Emerging evidence have shown that restoring SCFA-producing microbiota in aged mice could help to improve the post-stroke recovery, especially in the aspects of functional and cognitive impairments [46]. A study reviewed that SCFAs act as the ATP source for intestinal epithelial cells and improve the immune defensive functions of the intestinal epithelium. Butyrate produced by *Odoribacter* promoted the anti-inflammatory effect by impairing the lipopolysaccharide-induced NF-κB activation [45], which helps to reduce the ischemic injury by up-regulating the IRF3 activity [47].

However, an enrichment of lipopolysaccharide synthesis was observed in stroke cases as compared to healthy controls [15,17], and the presence of LBP can increase the rate of sepsis in stroke mice [6]. The bacterial lipopolysaccharide is a bacterial endotoxin, and it is an inflammatory marker found in the cell wall of Gram-negative microbiota. Emerging studies show that bacterial lipopolysaccharide is associated with ischemic stroke. An exposure to a small amount of lipopolysaccharide (100 μg/kg/dose) could significantly worsen the neurological deficits after stroke [48], while it could cause metabolic endotoxemia that triggered an inflammatory response among the obese and diabetic individuals [49]. An innate immune response could be triggered when a higher concentration of lipopolysaccharide reduced the expression of NF-κB subunit p65. This phenomenon reduces the release of cytokines to external stimulus and leads to apoptosis of macrophages, which subsequently causes the host body to be vulnerable to ischemic stroke due to low inflammatory response to bacterial infection [50].

Of the eligible studies, 55.6% reported on the translocation of microbiota from the gut to mesenteric lymph nodes, spleens, livers and lungs after ischemic stroke, which suggested an alteration of microbial composition in the body that subsequently affected the functional recovery of ischemic stroke [3–6,15,16,18,19,23,26]. Ischemic stroke tends to reduce gut barrier permeability of both human and mouse, in order to allow microbial trafficking into peripheral tissues and the brain [3,19]. The changes in the microbial composition disrupt the balance in gut immune homeostasis. Along the lines of inflammatory responses, lymphocytes were also observed to be migrated from the intestine to the brain after stroke [4,5], leading to a change in the abundance of inflammatory cells in the body site. The present study found that the genera *Clostridium*, *Lactobacillus*, and *Bacteroides* were present more abundantly in ischemic stroke after dysbiosis. These three microbiota are crucial in maintaining the immunological balance as they help to promote the growth of Treg cells to facilitate anti-inflammatory response through SCFAs production [51]. A recent study also showed that an immune response was activated after ischemic stroke through the crosstalk between M1 and Th1/Th17 cells and led to brain injury [47]. On the other hand, an anti-inflammation which was promoted by the crosstalk between M2 and Th2/Treg cells enhanced the recovery of the brain [52].

It is possible that there may be additional studies that were not identified, as the present systematic review found only 18 eligible studies. The scarcity of the literature resulted in most of the discussion are relied on animal studies, which have their own limitations. Each of the mice studies included used the homologous method in stroke induction. However, there are variations among them, which may introduce some selection bias. Besides, one of the limitations of this systematic review is that only nine clinical studies were included. The samples size in the clinical studies were different, ranging from 10 to 349 stroke cases. This condition may affect the representation of the present study to reflect a true population of ischemic stroke. Finally, the selection of hypervariable region used for 16s rRNA amplicon sequencing varied across both animal and clinical studies, and it may lead to the risk of amplification bias.

The Firmicutes/Bacteroidetes ratio is suggested to be a hallmark for aging and it is significantly associated with ischemic stroke in mouse models. From a clinical perspective, the assessment of Firmicutes/Bacteroidetes ratio in the human gut microbiome is highly recommended. Considering the differences in prevalence, the clinical findings of human microbiome should be stratified based on geographical, ethnicity, and gender, in order to distinguish the differences between these groups. Another concept that needs further exploration in clinical studies is the relationship between the dysbiotic microbiota and specific inflammatory markers that are present in the stroke mice. As SCFAs and TMAO have been shown as the emerging factors in ischemic stroke in animal studies, further studies investigating the association between SCFAs as well as TMAO among patients are required. In addition, there was only one animal study that raised the impact of antibiotics on modulation of microbiota in ischemic stroke treatment. More animal studies are needed to validate the impact of antibiotics in microbiome modulation for ischemic stroke treatment. Clinical studies are also encouraged to determine the impact of antibiotics treatment in the microbiome for post-stroke recovery. Nonetheless, the current study still serves as the complete guide for future ischemic stroke research.

## Conclusion

Human microbiome possesses a great potential in clinical implications of ischemic stroke. This systematic review provides clear insights on the association between human microbiome and ischemic stroke. In short, this is the first study that showed aging and inflammation may contribute to different microbial compositions and predispose individuals to ischemic stroke. The modulation of Firmicutes/Bacteroidetes ratio may be a potential target for therapeutic treatment of ischemic stroke.

## Supporting information

S1 Checklist PRISMA 2009 checklist. (DOC)
